# Supplementary figures and images for: Activation of the Drosophila innate immune system accelerates growth in cooperation with oncogenic Ras
Source: PLoS Biol. 2025 Apr 28;23(4):e3003068. doi: 10.1371/journal.pbio.3003068 (PMC12036928; doi:10.1371/journal.pbio.3003068)

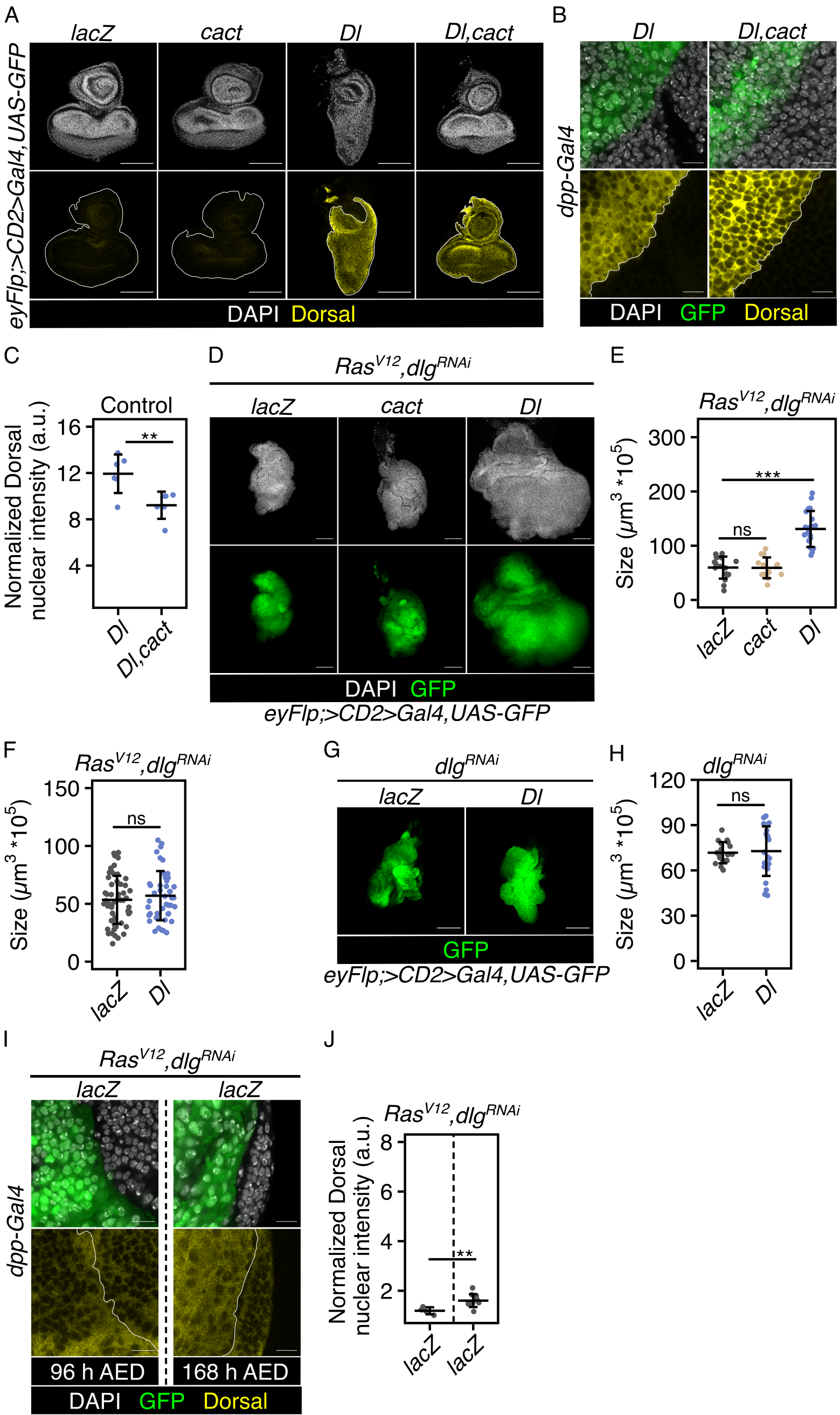

Supplement: S1 Fig — (A) Confocal images of EADs of indicated genotypes at 96-h AED stained for Dorsal (yellow) to illustrate the efficiency of overexpressing Cactus to block the morphological defects caused by Toll signaling activation by Dorsal overexpression. The white line highlights the GFP-marked region. (B) Representative confocal images of regions of wing discs (WDs) of indicated genotypes at 96-h AED stained for Dorsal (yellow). Nuclear Dorsal levels are quantified in (C). The white line highlights the border between GFP-marked cells of the Dpp stripe and GFP-negative cells of the posterior WD compartment. (C) Quantification of Dorsal nuclear intensity of the indicated genotypes. The mean fluorescence intensity of Dorsal in GFP-positive cells, internally normalized to GFP-negative cells, and standard deviation (error bar) are shown (**p < 0.01, One-way ANOVA with post-hoc Tukey HSD). (D) Confocal images of RasV12, dlgRNAi-induced EAD tumors of indicated genotypes at 120-h AED representative for tissue sizes quantified in (E). (E) and (F) Quantification of RasV12, dlgRNAi-induced tumor volume of indicated genotypes at 120-h AED (E) and 96-h AED (F). Mean volume and standard deviation (error bar) are shown (***p < 0.001, ns: p = 0.99 (E) or ns: p = 0.41 (F), One-way ANOVA with post-hoc Tukey HSD). (G) Confocal images of dlgRNAi-transformed EADs of indicated genotypes at 120-h AED representative for tissue sizes quantified in (H). Images are extracted singles planes of samples mounted for 3D quantification. (H) Quantification of dlgRNAi-transformed EAD volume of indicated genotypes at 120-h AED. Mean volume and standard deviation (error bar) are shown (ns: p = 0.8, One-way ANOVA with post-hoc Tukey HSD). (I) Representative confocal images of regions of WDs of indicated genotypes at 96-h AED (or 168-h AED) stained for Dorsal (yellow). Nuclear Dorsal levels are quantified in (J). The white line highlights the border between GFP-marked cells of the Dpp stripe and GFP-negative cells o [file pbio.3003068.s001.tif]

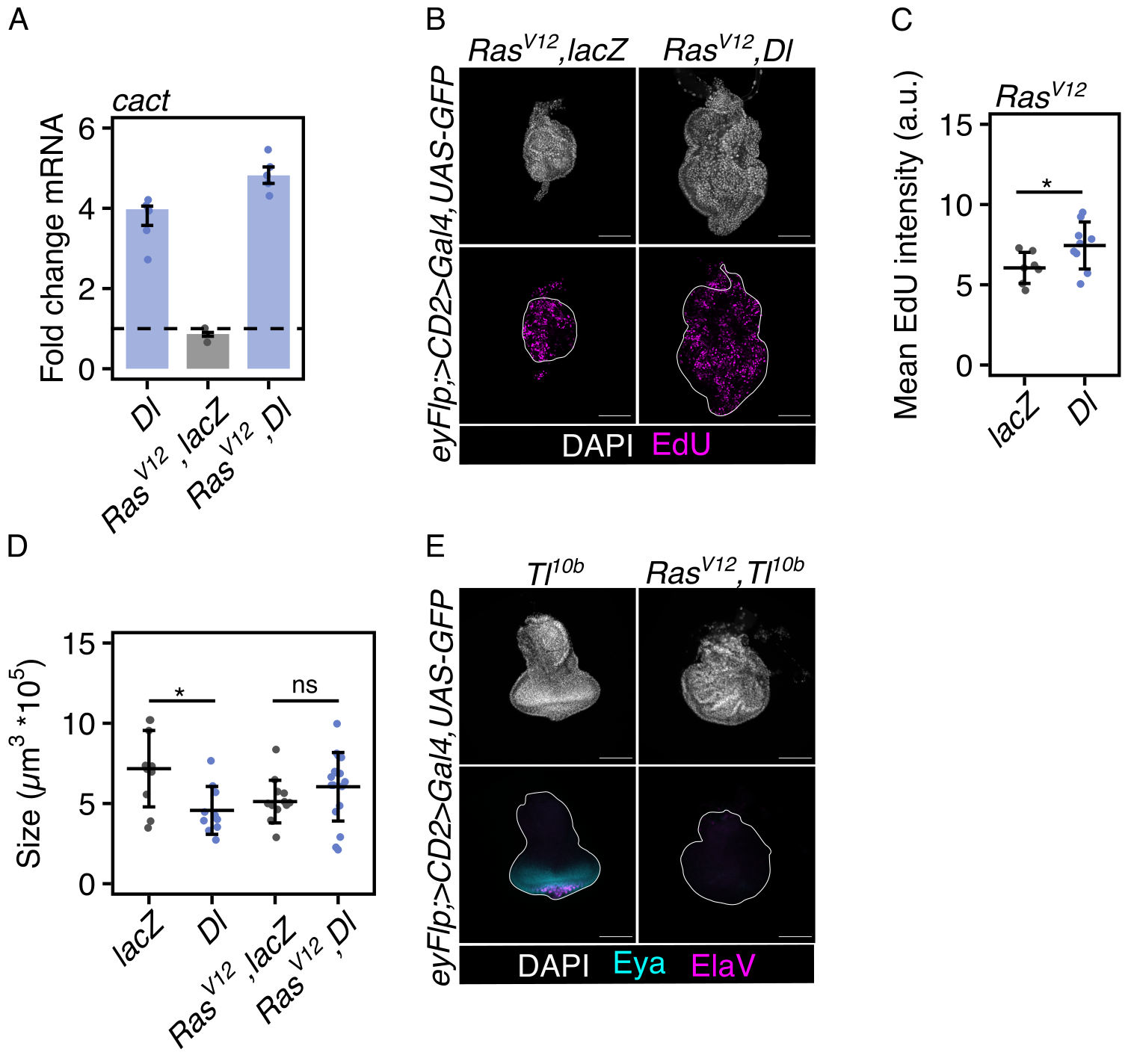

Supplement: S2 Fig — (A) qRT-PCR analysis of cact mRNA levels in EADs expressing indicated UAS-transgenes under the control of an eye-specific Gal4 (eyFlp; act>CD2>Gal4) as a read-out for Toll signaling activity. Data is shown as fold changes relative to the control (lacZ). n = 3 biologically independent samples were analyzed. Median fold changes and interquartile ranges (error bars) are shown. The dashed horizontal line illustrates the reference fold change of 1. (B) Confocal images of EAD tumors of indicated genotypes at 96-h AED stained with Click-iT EdU Alexa Fluor 647 (magenta) to identify DNA synthesis-based proliferation representative for EdU intensity quantified in (C). The white line highlights the GFP-marked region. (C) Quantification of EdU mean fluorescence intensity per EAD tumor at 96-h AED. Mean intensity and standard deviation (error bar) are shown (*p < 0.05, One-way ANOVA with post-hoc Tukey HSD). (D) Quantification of tissue volume of indicated genotypes at 72-h AED illustrating the time-dependency of Dorsal-induced tumor overgrowth. Mean volume and standard deviation (error bar) are shown (*p < 0.05, ns: p = 0.55, One-way ANOVA with post-hoc Tukey HSD. (E) Confocal images of an EAD and tumor of the indicated genotype at 96-h AED labeled for retinal determination and photoreceptor differentiation markers Eya (cyan) and ElaV (magenta). The white line highlights the GFP-marked region. In all confocal images, DAPI (gray) is used to visualize nuclei and GFP (green) labels cells co-expressing indicated UAS-transgenes. The underlying data sets can be found in S4 Data. Scale bars represent 100 µm. ns, not significant; a.u., arbitrary unit; EAD, eye-antennal disc; AED, after egg deposition; D,: Dorsal; Tl10b, Toll10b; eyFlp, eyeless-driven Flippase; Eya, eyes absent; ElaV, embryonic lethal abnormal vision; EdU, 5-ethynyl-2′-deoxyuridine. (TIF) [file pbio.3003068.s002.tif]

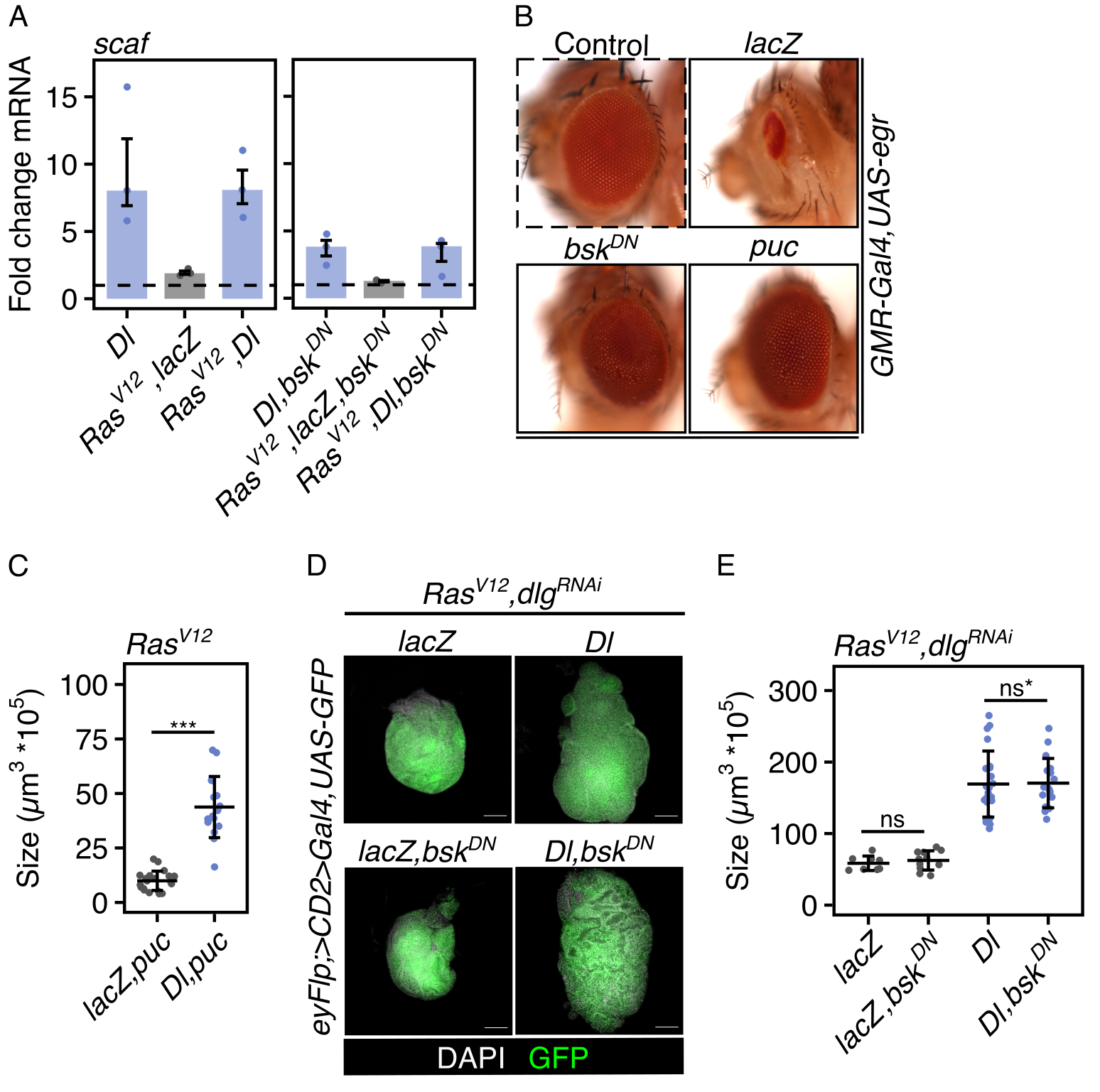

Supplement: S3 Fig — (A) qRT-PCR analysis of scaf mRNA levels in EADs expressing indicated UAS-transgenes under the control of an eye-specific Gal4 (eyFlp; act>CD2>Gal4) as exemplary confirmation of the JNK-dependent upregulation of target genes after overexpression of Dorsal. Data is shown as fold changes relative to the control (lacZ or lacZ,bskDN). n = 3 biologically independent samples were analyzed. Median fold changes and interquartile ranges (error bars) are shown. The dashed horizontal line illustrates the reference fold change of 1. (B) Images of adult eyes of indicated genotypes showing the efficiency of overexpressing BskDN or Puc to inhibit JNK signaling and rescue Egr induced loss of photoreceptor cells. All flies, except for wild-type control flies expressed GMR-Gal4, UAS-egr. (C) Quantification of tumor sizes of indicated genotypes at 96-h AED confirming the observation of JNK-independent induction of tumor overgrowth after Dorsal overexpression using co-expression of Puc to inhibit JNK signaling. Mean volume and standard deviation (error bar) are shown (***p < 0.001, One-way ANOVA with post-hoc Tukey HSD). (D) Confocal images of of EADs and EAD tumors at 96-h AED of the indicated genotypes representative for tissue sizes quantified in (E). The white outline highlights the GFP-marked region. (E) Quantification of tissue volume of indicated genotypes at 120-h AED illustrating that growth of RasV12, dlgRNAi tumors did not depend on JNK signaling at 120-h AED. Mean volume and standard deviation (error bar) are shown (ns: p = 0.56, ns*: p = 0.92, One-way ANOVA with post-hoc Tukey HSD). In all confocal images, DAPI (gray) is used to visualize nuclei and GFP (green) labels cells co-expressing indicated UAS-transgenes. The underlying data sets can be found in S6 Data. ns, not significant; EAD, eye-antennal disc; AED, after egg deposition; Dl, Dorsal; mmp1, matrix metalloproteinase 1; ets21c, Ets at 21c; bskDN, dominant-negative Basket; Puc, Puckered; GMR, glass multiple reporter [file pbio.3003068.s003.tif]

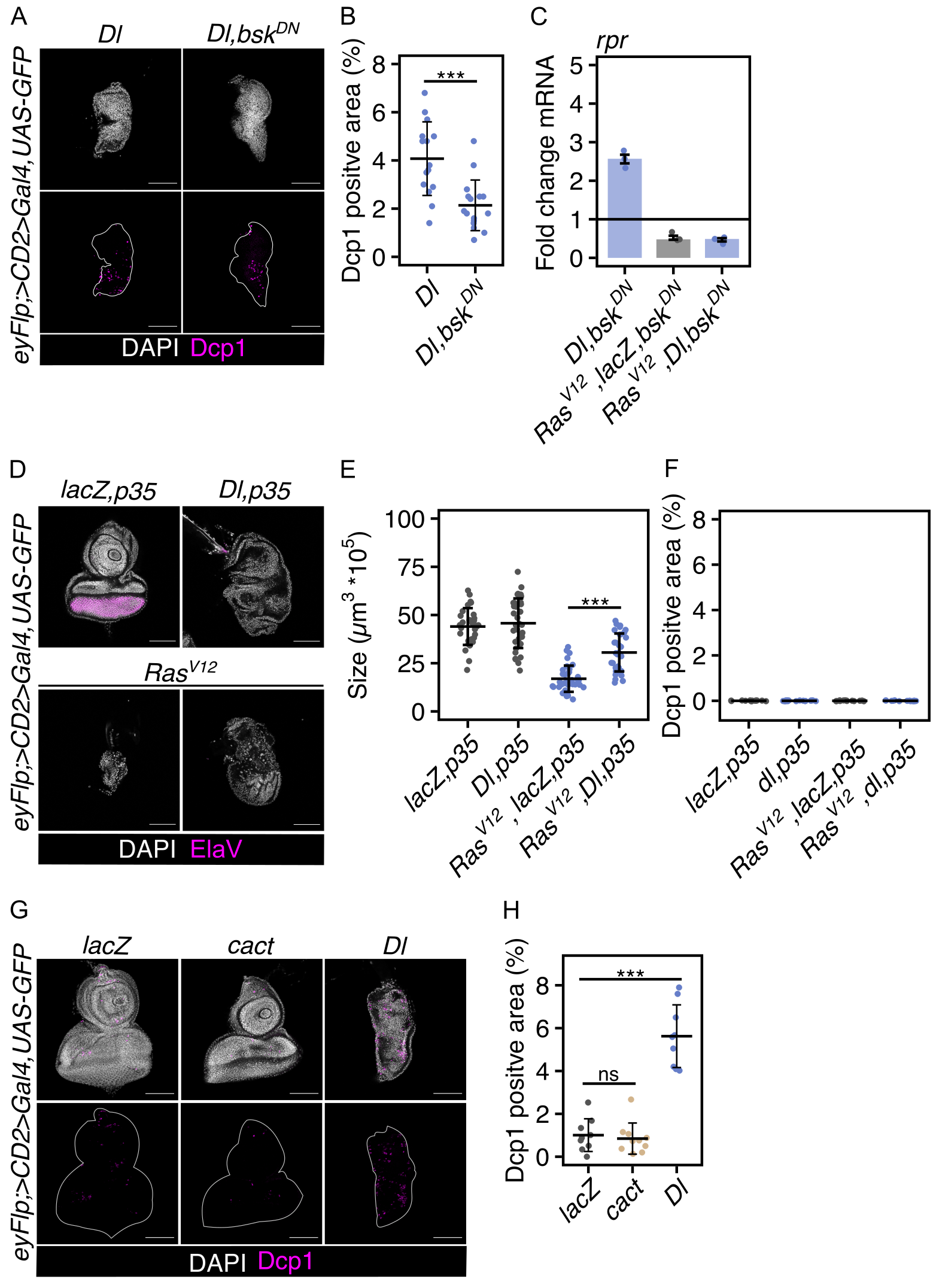

Supplement: S4 Fig — (A) Confocal images of EADs of indicated genotypes at 96-h AED labeled for Dcp1 showing the persistence of elevated levels of Dcp1 after JNK inhibition. The white outline highlights the GFP-marked region. (B) Quantification of the Dcp1-positive area relative to whole disc size at 96-h AED comparing control EADs after overexpression of Dorsal with BskDN. Mean area and standard deviation (error bar) are shown (***p < 0.001, One-way ANOVA with post-hoc Tukey HSD). (C) qRT-PCR analysis of rpr mRNA levels in EADs expressing indicated UAS-transgenes under the control of an eye-specific Gal4 (eyFlp; act>CD2>Gal4) illustrating the JNK-independent upregulation of pro-apoptotic gene expression after overexpression of Dorsal in control EADs. Data is shown as fold changes relative to the control (lacZ, bskDN). n = 3 biologically independent samples were analyzed. Median fold changes and interquartile ranges (error bars) are shown. The dashed horizontal line illustrates the reference fold change of 1. (D) Confocal images of EADs of indicated genotypes at 96-h AED labeled for ElaV to highlight the lack of photoreceptor cells after Dorsal and p35 co-expression. (E) Quantification of tissue volume of indicated genotypes at 96-h AED to confirm the increased growth of discs overexpressing Dorsal after inhibition of apoptosis by co-expression of p35 using three-dimensional quantification. Mean volume and standard deviation (error bar) are shown (***p < 0.001, One-way ANOVA with post-hoc Tukey HSD). (F) Quantification of the Dcp1 positive area relative to whole disc size at 96-h AED. Mean area and standard deviation (error bar) are shown. (G) Confocal images of EADs of indicated genotypes at 96-h AED labeled for Dcp1 confirming there is no upregulation of cell death after overexpression of cactus. The white outline highlights the GFP-marked region. (H) Quantification of the Dcp1-positive area relative to whole disc size at 96-h AED comparing cell death levels in control EADs after over [file pbio.3003068.s004.tif]
